# Towards high-quality development: The complex role of environmental regulation

**DOI:** 10.1371/journal.pone.0312816

**Published:** 2025-02-12

**Authors:** Jie Li, Xuguang Wang

**Affiliations:** 1 School of Law, Xuchang University, Xuchang, China; 2 School of Business, Xuchang University, Xuchang, China; Universita degli Studi del Molise, ITALY

## Abstract

High-quality development is a necessary condition for a healthy society, and environmental regulation is an important means to maintain high-quality development. Against this backdrop, this study takes the Yellow River Basin in China as an example to explore the complex impact of environmental regulation on high-quality economic development, with a particular focus on the indirect impact of regional pollution heterogeneity on the complex influence. Utilizing panel data from 2011 to 2022, this paper constructs an evaluation index system for the Yellow River Basin’s high-quality development that encompasses 29 fundamental indicators and applies the Entropy-Weighted TOPSIS method for a comprehensive assessment. The research found a significant positive relationship between environmental regulation and high-quality economic development, confirming the key role of environmental regulation in balancing economic growth and environmental protection. The article further analyzes the complex impact of environmental regulation under the background of pollution heterogeneity and discovers the nonlinear and threshold effect characteristics between environmental regulation and high-quality development. Notably, the positive influence of environmental regulation on high-quality economic development is already evident at lower intensities, yet becomes markedly more pronounced when the regulatory intensity reaches or exceeds a certain threshold. Additionally, the paper delves into the heterogeneous impacts of environmental regulation on high-quality economic development across regions with differing pollution levels. The findings indicate that in regions of severe pollution, the positive effect of environmental regulation is considerably more pronounced, potentially attributable to the heightened public concern for environmental issues and the stringent environmental protection measures implemented by the government. The study also addresses the issue of endogeneity using instrumental variable methods to ensure the robustness of the results. Ultimately, the paper presents targeted policy recommendations, including the formulation of region-specific environmental regulations, the establishment of regional collaborative governance mechanisms, the enhancement of public participation and oversight, and the promotion of green technological innovation, all aimed at fostering the coordinated advancement of high-quality economic development and environmental protection in the Yellow River Basin.

## 1 Introduction

Since the initiation of reforms and the opening up of its economy in 1978, China has embarked on a phase of rapid industrialization and economic expansion. Arriving at the year 2023, the nation’s GDP has soared to a staggering 126 trillion yuan, with its manufacturing sector contributing nearly 30% to the global industry, effectively positioning itself as the central force driving global industrial growth. Over the past 40-plus years, the rapid growth of China’s economy has been mainly attributed to the continuous increase in traditional factors of production [1]. As per capita income continues to climb, China is progressively ascending into the ranks of high-income nations, with its economic share on the global stage consistently rising, augmenting its international influence significantly. However, this growth is shadowed by significant environmental pollution and emissions, posing a threat to the sustainability of economic achievements [2]. The traditional growth model, characterized by substantial input of production factors, has become obsolete in the face of contemporary development demands [3]. The exacerbation of environmental issues not only curbs sustained economic growth but also impinges upon the quality of life and the well-being of the populace. Confronted with these challenges, China is compelled to pivot its development paradigm, enhance economic efficiency, and pursue a trajectory of high-quality development. The Chinese government has come to fully recognize this imperative, articulating strategic responses within various national-level development plans. These include initiatives to catalyze green transformation [4], achieve high-quality development [5], set goals for carbon peak and carbon neutrality [6], and establish a carbon emission trading market [7]. Underpinning these policies, environmental regulation plays an indispensable role, and its capacity to foster equilibrium between economic expansion and environmental preservation has emerged as a focal point within academic research.

The salient features of environmental issues are the negative externalities of environmental pollution and the public goods nature of environmental products. Environmental regulation is an important measure to balance environmental issues [8]. Environmental regulation represents an array of policies and measures enacted by governments to ameliorate environmental pollution and ecological degradation. Its fundamental purpose is to intervene and modulate the environmental negative externalities potentially generated by economic activities through legal, administrative, and economic avenues, thereby striving for a harmonious coexistence of economic and environmental development [9]. The genesis of environmental regulation dates back to the Industrial Revolution. At the turn of the 20th century, select Western nations initiated legislation to curb industrial pollution. These nascent environmental regulations centered on direct control of pollutant emissions, employing a command-and-control approach that mandated enterprises to adhere to specific emission standards. Advancing into the mid-to-late 20th century, against a backdrop of escalating environmental concerns and an upsurge in public environmental consciousness, environmental regulation began to evolve towards diversification, with the emergence of market incentive-based regulatory instruments. In tandem with the march of globalization, environmental regulation has also begun to exhibit international dimensions. International accords such as the 1992 United Nations Framework Convention on Climate Change and the 1997 Kyoto Protocol signify the cooperation and coordination of environmental regulation at a global scale. China’s foray into environmental regulation, albeit delayed, has been rapid. Post the economic reforms, with the swift economic ascent, the Chinese government commenced prioritizing environmental concerns, instituting a suite of environmental protection laws and regulations. As the 21st century unfolded, China’s environmental regulation has increasingly emphasized a fusion of prevention and management, propelling the progression towards green development and the construction of ecological civilization.

The Yellow River Basin, an expansive geographical and ecological region stretching from the river’s origins to its delta, encompasses nine provinces: Qinghai, Sichuan, Gansu, Ningxia, Inner Mongolia, Shaanxi, Shanxi, Henan, and Shandong. The Yellow River Basin spans across the eastern and western parts of China, encompassing provinces that are economically developed as well as those that are relatively underdeveloped economically. This basin is pivotal as a significant base for China’s energy, chemical industry, and raw materials sectors. It is not only a vital ecological shield for the nation but also an essential economic belt. The high-quality development of the Yellow River Basin is a strategic imperative for China, evolving into a critical growth pole for regional development in contemporary times and a principal arena for the country’s developmental elements. Presently, the provinces and regions within the Yellow River Basin are contending with formidable challenges such as expanding disparities in regional economic development, escalating energy consumption, waning momentum for high-quality economic development, and pollution afflicting air, water, and solid waste. To transcend the constraints imposed by resources and the environment and to propel the economy of the Yellow River Basin towards loftier echelons of quality, identifying and capitalizing on pivotal developmental nodes and novel opportunities bear significant practical implications for the robust growth of China’s aggregate economy and the burgeoning economies on the global stage.

Industrial pollution stands as a principal driver of environmental degradation in the Yellow River Basin [10, 11], and the deployment of environmental regulations is a universal strategy to reconcile economic advancement with ecological conservation [12–14]. In pursuit of fostering high-quality development, the provinces along the Yellow River have enacted a suite of environmental regulations. In June 2022, the Chinese government promulgated the Yellow River Basin Ecological Environment Protection Plan, with the goal of enhancing the region’s ecological quality. Nonetheless, challenges persist, including inadequate regulatory enforcement, a blanket policy approach, and insufficient oversight, which hinder the effective control of corporate pollutant emissions. Consequently, devising tailored environmental strategies that reflect the diverse pollution levels within the Yellow River Basin is essential for propelling the region’s high-quality development. Current scholarly work predominantly centers on the interplay between environmental regulations and economic growth, with the nexus between environmental regulations and high-quality development receiving nascent attention. The establishment of a systematic framework for the assessment and evaluation of high-quality development remains an evolving field, marked by a diversity of approaches to constructing its indicators. Furthermore, the selection of research samples and timeframes can significantly shape research outcomes. Pronounced variations exist across Chinese provinces and regions, particularly in the central and western areas, in terms of resource endowments, population demographics, economic growth patterns, and the composition of energy and industry. The Yellow River Basin, extending over multiple provinces and encompassing these diverse attributes, presents an optimal natural laboratory for scrutinizing how pollution heterogeneity influences the impact of environmental regulations on high-quality development.

The paper’s marginal contributions are threefold: First, it constructs a high-quality development evaluation index system for the Yellow River Basin, integrating 29 foundational indicators across the dimensions of innovation, coordination, green practices, openness, and sharing, in line with China’s strategic development philosophy. It applies the Entropy-Weighted TOPSIS method for a comprehensive assessment of the development quality in the sampled areas. Second, it adopts a novel perspective that considers the heterogeneity of pollution levels, rather than just spatial heterogeneity, to examine the diverse impacts of environmental regulation on high-quality development. Third, it refines regional environmental policies from the vantage point of pollution heterogeneity, providing fresh evidence for formulating environmentally tailored regulations in various regions, thereby offering scientific guidance for emerging economies globally in the precise development of environmental policies and the efficient advancement of high-quality development.

## 2 Literature review and theoretical hypotheses

### 2.1 Literature review

#### 2.1.1 Environmental regulations

In the economically challenged 1980s, China designated environmental protection as a cornerstone of its national policy. The landmark legislation, "Environmental Protection Law of the People’s Republic of China," was enacted in 1989, outlining regulations for environmental conservation, oversight, pollution control, and legal accountability. However, economic growth is the primary goal of the traditional development perspective; if this view does not change, environmental issues will continue to worsen. A revised edition of the law was introduced in 2015, expanding the legal framework to include 29 environmental statutes, such as the "Water Pollution Prevention Law" and the "Atmospheric Pollution Prevention Law," among others [15]. The natural environment, being the foundation for all human activities, including economic endeavors, has a profound and varied influence that has drawn the interest of scholars across various disciplines. Researchers such as Ambec et al. [16] have dissected the interplay between environmental regulation and the Porter Hypothesis through the lens of policy reform, establishing a research framework for contemporary studies in this domain. Rubashkina [17] delved into the nexus between environmental regulation and corporate innovation and productivity, uncovering a significant catalytic effect on innovation, albeit with limited impact on productivity. Yenipazarli [18] utilized the Stackelberg game theory to examine the influence of emission tax policies on manufacturers’ optimal production and pricing strategies, highlighting how strategic business planning under environmental regulation can curb pollution while boosting profits. López-Gamero [19] explored the dynamic between environmental regulation and proactive environmental management, revealing that while mandatory regulations have a muted effect, voluntary measures significantly enhance management practices and, in turn, corporate competitiveness. This esteemed body of literature, with a focus on enterprises and employing a blend of empirical and normative research methodologies, underscores that environmental regulation has complex impacts on the economy, society, and nature. Furthermore, it illustrates the interdisciplinary nature of environmental regulation, offering a rich tapestry for future academic inquiry.

#### 2.1.2 High-quality development

In 2017, the concept of high-quality development was first articulated in China, and since then, it has gained prominence in scholarly and policy discourse. This term is often employed to denote an economic growth paradigm that transcends the mere pursuit of pace and magnitude, focusing instead on the quality and efficacy of growth. It underscores the importance of refining economic structures, bolstering innovation as a driving force, optimizing the allocation of resources, ensuring the sustainability of environmental conservation, and significantly elevating social welfare. Despite the concept’s apparent simplicity, its precise delineation, assessment of advancement, and practical implementation present a tapestry of intricate theoretical and practical challenges.

Chinese scholars, in their papers published in Chinese, have extensively focused on the background, connotations, characteristics, and implementation pathways of high-quality development. Jin [20] posits that high-quality development possesses notable dynamic and multidimensional attributes. The new concepts of development that emphasize innovation, coordination, green initiatives, openness, and sharing serve as the criteria for evaluating high-quality development. A modern governance system is required to guide the realization of high-quality development. The assessment of high-quality development must exhibit systemic characteristics; the goals and approaches of high-quality development are dynamic, and the process of achieving it is characterized by long-term features [21, 22]. High-quality economic development can address structural imbalances in China’s economy and enhance total factor productivity [23]; the realization of high-quality development necessitates impetus from the perspectives of systemic reform and mechanism transformation [24]; equitable income distribution [25], financial services [26], a smart economy [27], and the enhancement of human capital [28] are vital avenues for achieving high-quality development.

Despite an abundance of scholarly work on the high-quality economic development, a cohesive theoretical framework and index system for its quantification remain elusive. The literature can be primarily divided into two categories: single-indicator and composite-indicator approaches. The single-indicator method evaluates economic development quality through one metric, typically total factor productivity [23] or GDP per capita [29]. Conversely, the composite-indicator approach constructs an index system grounded in the five key developmental concepts [30, 31]. Considering the potential for a myopic view and resultant biases inherent in the single-indicator method, the majority of the current literature favors the more holistic composite-indicator methodology.

#### 2.1.3 Relationship between environmental regulation and high-quality development

Within the academic sphere, the discourse on the interplay between environmental regulation and economic development is both expansive and multifaceted. It spans a vast array of disciplines and subjects, encompassing macroeconomic strategies and microeconomic corporate operations, as well as specific industry analyses and cross-regional comparisons. These scholarly endeavors have not only examined the immediate effects of environmental regulation on economic growth but have also provided in-depth analyses of its broader implications for corporate innovation, the restructuring of industrial sectors, and the enhancement of social welfare. Consequently, the conclusions are varied, mirroring the intricate and multifaceted nature of the subject [32–34]. When it comes to the nexus between environmental regulation and the high-quality development of the economy, academia is divided into three predominant viewpoints:

1. Some scholars believe that environmental regulation can stimulate the innovation capability of enterprises, that is, stimulate the "innovation effect", thereby improving production efficiency and promoting economic growth, which is known as the "Porter Hypothesis" [35].By establishing stringent pollution emission standards and requirements, environmental regulation compels businesses to seek more efficient and eco-friendly production methods. This external pressure often catalyzes a company’s innovative potential, encouraging investment in research and development (R&D) to create new technologies and products that meet regulatory demands [36]. This process not only fosters an increase in corporate production efficiency but also, through innovation, leads to the development of new products and services [37] that can break into markets and create new growth opportunities [38]. Moreover, environmental regulation can enhance resource utilization efficiency, reduce long-term operational costs, and strengthen a company’s market competitiveness. As corporate innovation capabilities and production efficiencies improve, the entire industry and even the national economy can reap benefits, achieving sustainable development and economic growth [35]. The Porter Hypothesis suggests that appropriate environmental regulation can motivate companies to engage in technological innovation, thereby gaining a competitive edge [39]. Jaffe and Palmer expanded on the Porter Hypothesis, delineating the narrow, weak, and strong Porter Hypotheses [40]. Numerous scholars have conducted a series of tests around this hypothesis. Early studies, such as those by Henriques et al. [41], proposed that formal environmental regulations can positively promote the efficiency of corporate green innovation. Recent research, including studies by Li et al. [42] and Santis et al. [43], also supports the Porter Hypothesis, asserting that environmental regulation drives technological innovation, which in turn benefits economic development.
2. A different group of scholars argues that environmental regulation increases the costs for businesses, leading to a decrease in production efficiency and thus adversely affecting economic development [44, 45], which belongs to the "cost effect" school of thought [46]. These costs encompass, but are not limited to, investments in pollution control technologies, the purchase and maintenance of environmental protection equipment, and the acquisition of pollution emission rights. Such additional expenditures may compress the profit margins of businesses, affecting their ability to reinvest and expand [40]. The "compliance cost theory" in neoclassical economics posits that these costs from environmental regulation will lead to a decline in business production efficiency, as companies must balance meeting regulatory requirements with maintaining competitiveness. This perspective holds that if environmental regulations are too stringent, they may suppress business innovation and expansion, thereby negatively impacting overall economic development. Furthermore, for smaller businesses with weaker competitiveness, environmental regulations could become a barrier to their growth, potentially causing some businesses to exit the market. Therefore, finding a balance between protecting the environment and promoting economic development is a critical issue that policymakers need to consider carefully.
3. A third perspective posits that the relationship between environmental regulation and high-quality economic development is not linear but exhibits a certain curvilinear pattern. In the early stages of environmental regulation implementation, the "suppression effect" of costs predominates. However, once the intensity of regulation surpasses a certain threshold, the compensatory effect of innovation begins to take over, creating an U-shaped relationship. Researchers have delved into the multiple impacts of environmental regulation on the economy and environment through different datasets and models. For instance, Wang et al. [47], by analyzing panel data from the industrial sectors of OECD member countries and using an extended SBM-DDF method to measure the stringency of environmental regulation, found that within a certain range of policy enforcement strictness, environmental policies can actively promote the growth of green productivity. However, when the strictness of policy exceeds a certain threshold, the increase in compliance costs begins to outweigh the offsetting effects of innovation, leading to a negative impact on the growth of green productivity. This finding supports the strong version of the Porter Hypothesis. Song’s [48] research re-examined the relationship between the intensity of environmental regulation and the rate of economic growth. Based on panel data from 31 provinces and cities in China from 2004 to 2017, Song found a nonlinear "inverted U-shaped" relationship between environmental regulation and the rate of economic growth, where the rate of economic growth initially rises with the increase in the intensity of environmental regulation and then turns to decline, indicating that the internal impact pathways of environmental regulation vary with different types of industries. Furthermore, Zhang et al. [49] constructed a dynamic spatial panel model for China’s provinces from 2000 to 2017 to evaluate the nonlinear and spatial effects of environmental regulation on economic growth and carbon dioxide emissions. Their research results reveal that at the national level and in the eastern and central regions, the impact of environmental regulation on economic growth shows a significant "N-shaped" effect, while the impact on carbon dioxide emissions is an "inverted N-shaped." In contrast, both economic growth and carbon dioxide emissions in the western region show "N-shaped" effects. Additionally, the intensification of regional environmental regulation has a positive spillover effect on the reduction of economic growth and carbon dioxide emissions in neighboring provinces, but in the western provinces, this spatial effect shows an opposite trend. Therefore, technological innovation does not guarantee a synchronized enhancement of economic development and environmental quality [50]. Technological innovation has the potential to both increase and decrease pollution, thus necessitating a coordination of technological innovation and economic development through environmental regulation [51, 52].

Reviewing the academic discourse on the relationship between environmental regulation and green economic development, the research indicates a complex, nonlinear relationship between environmental regulation and high-quality economic development. Heterogeneity is observed across different industries, stages, and regions, potentially related to different stages of the Porter Hypothesis, which affects the efficacy of environmental regulation. Discrepancies in measurement methods also lead to divergent results. Research must take into account the characteristics of economic entities, such as green development, technological advancement, and industrial upgrading in the Yellow River Basin. The wide-ranging conclusions necessitate further in-depth study. Most existing research is based on spatial heterogeneity, neglecting the issue of pollution heterogeneity. Starting from the perspective of pollution heterogeneity can more comprehensively reflect geographical attributes, enhancing the scientific and practical significance of the research, aiding in a deeper understanding of the relationship between environmental regulation and high-quality economic development, and providing theoretical support for policy-making.

### 2.2 Theoretical analysis and assumptions

#### 2.2.1 Linear relationship

The government acts as the principal enforcer of environmental regulation policies, typically enhancing the stringency of these policies by increasing the environmental costs for businesses—such as through the imposition of environmental taxes. From the perspective of innovation incentives, well-crafted environmental regulations can stimulate technological advancement and industrial upgrading. The Porter Hypothesis suggests that stringent yet appropriate environmental regulations can spur corporate innovation, with businesses developing new technologies and improving production processes to increase resource efficiency and reduce pollution emissions. As an external pressure, environmental regulations compel companies to seek innovative solutions to meet environmental standards, thereby enhancing their market competitiveness and economic benefits. This innovation-driven mechanism not only aids businesses in breaking through with environmentally friendly technologies but also fosters the upgrading of the entire industry chain and the optimization of economic structures, ultimately promoting high-quality economic development.

From the perspective of market effects, appropriate environmental regulation can guide the allocation of market resources towards a green economy and sustainable development. By internalizing external costs, environmental regulations make businesses bear the true cost of their environmental impact, encouraging the market to favor environmentally friendly enterprises and industries. This optimization of resource allocation helps to develop a low-carbon economy and green industries, reducing environmental pollution and resource waste. Under the guidance of suitable environmental regulations, market mechanisms can create demand for green products and technologies, promoting the development of the environmental protection industry and enhancing the quality of economic growth. In the long run, this market effect will continue to drive the economy towards sustainable and high-quality development.

From the perspective of social benefits, appropriate environmental regulation can promote the improvement of social welfare and public health, thereby indirectly promoting high-quality economic development. Environmental regulations enhance environmental quality and living standards by controlling pollution and protecting natural resources, reducing the incidence of environment-related diseases and public health expenditure. This enhancement of social benefits not only improves the health and productivity of the workforce but also reduces social medical costs and raises the overall level of social welfare. As social welfare improves, the quality and sustainability of economic development will also be strengthened. Therefore, appropriate environmental regulation indirectly promotes high-quality economic development by enhancing social benefits. Based on the above theoretical analysis, Hypothesis 1 is proposed:

**H1**: **Appropriate environmental regulation has a promoting effect on high-quality economic development.**

#### 2.2.2 Nonlinear relationship

Enterprises are the microeconomic agents responsible for achieving high-quality development. For businesses with varying levels of pollution discharge, the costs of pollution control and the impact of environmental regulation differ. There exists a dynamic equilibrium between the high initial investment costs and the subsequent benefits of innovation. When environmental regulations are first implemented, companies must invest substantial resources to comply with new standards, including research and development, equipment upgrades, and operational adjustments, which may lead to increased production costs and a decline in economic benefits in the short term. However, over time, these initial investments gradually translate into innovative outcomes. By developing new technologies and optimizing production processes, companies enhance their efficiency and market competitiveness, thereby achieving greater economic benefits and quality development. Thus, the impact of environmental regulation on economic development may exhibit a nonlinear characteristic that initially suppresses and then stimulates growth. Extending this analysis to the regional level suggests that the macroeconomic impact of environmental regulation in areas with heterogeneous pollution levels also has nonlinear characteristics.

Based on practical experience, there is a positive correlation between a company’s pollution control costs and the intensity of environmental regulation; as the stringency of environmental regulation increases, so do the pollution control costs for businesses. The cost for a company to reduce each unit of pollutant emissions tends to rise, indicating an increasing marginal cost of pollution control. This is both the source of rising environmental regulation costs and the basis for the nonlinear impact of environmental regulation on high-quality economic development. According to market economy theory, in the early stages of environmental regulation implementation, strict regulations may lead to some highly polluting, high-energy-consuming companies exiting the market, causing a short-term negative impact on economic growth. However, this market adjustment helps to phase out outdated production capacities and guide resources towards technologically advanced, environmentally efficient businesses and industries, promoting the optimization and upgrading of industrial structures. As market mechanisms adjust and resources are reallocated, the environmental protection industry and green economy will develop, propelling the economy into a new stage of high-quality development.

Additionally, initial environmental regulations may provoke resistance and opposition from some businesses and the public, primarily due to perceived loss of interests and increased adaptation costs. Such societal reactions could lead to obstacles in policy implementation and a weakening of its effects. However, as public awareness of environmental protection grows and the benefits of environmental regulation are recognized, societal acceptance will gradually increase, and the execution and efficacy of policies will be strengthened. Concurrently, governments and policymakers, based on initial feedback, continuously refine and adjust regulatory measures to make them more scientific and appropriate, ultimately achieving a win-win situation for environmental protection and economic development. The nonlinear interaction between societal response and policy optimization also reflects the nonlinear impact of environmental regulation on the transition to high-quality development. Based on the above theoretical analysis, Hypothesis 2 is proposed:

**H2**: **Environmental regulation has a nonlinear impact on high-quality development.**

#### 2.2.3 Heterogeneity

In severely polluted areas, where environmental issues are acute, the government and businesses have an increased urgency for technological innovation. Stringent environmental regulations encourage companies to increase their investment in research and development of environmental protection technologies and clean production processes, thereby achieving technological breakthroughs and industrial upgrading. Compared to areas with lighter pollution, regions with heavy pollution are more likely to generate a concentrated effect of technological innovation in the face of environmental regulation. This effect not only improves environmental quality but also enhances the quality and sustainability of economic growth. Therefore, the role of environmental regulation in promoting technological innovation in heavily polluted areas is more pronounced, which in turn promotes their high-quality development.

From the perspective of resource reallocation, heavily polluted areas often accumulate a plethora of inefficient and highly polluting enterprises. These businesses face significant compliance costs under stringent environmental regulations, which may lead to their shutdown or relocation. While this process can have a negative impact on economic growth in the short term, it is beneficial in the long run as it helps optimize resource allocation, phase out outdated production capacities, and foster the development of high-efficiency, low-pollution businesses. The economic structural adjustments and technological upgrades brought about by resource reallocation make the achievement of high-quality development in heavily polluted areas more pronounced under the impetus of environmental regulation.

From the point of societal and policy responses, in regions with severe pollution, there is heightened public concern for environmental issues and increased governmental pressure for environmental protection. This has driven the introduction and enforcement of stricter and more effective environmental regulation policies. Liu [53] believes that as China faces increasingly severe environmental issues, the public’s awareness and demand for environmental protection are also continuously growing; the high level of public concern about environmental issues significantly promotes the green development of cities; with the increased public attention to the environment, government financial intervention has also been strengthened, which further promotes the development of cities towards a greener and more sustainable direction. Governments implement more rigorous environmental measures and incentive policies in heavily polluted areas, such as environmental tax incentives and green subsidies, to encourage businesses to undertake environmental renovations and technological upgrades. The combined effect of these policy responses and societal pressures enables regions with significant pollution to achieve high-quality economic development more rapidly and markedly under environmental regulation. Based on the aforementioned theoretical analysis, Hypothesis 3 is proposed:

**H3**: **The impact of environmental regulation on high-quality economic development varies across regions with different levels of pollution, with a more pronounced effect in heavily polluted areas.**

## 3 Research design

### 3.1 Model and data source

#### 3.1.1 Model setting and variable description

This paper primarily investigates the impact of environmental regulation on the high-quality economic development of the Yellow River Basin, employing panel data regression methods for empirical testing. The level of high-quality development in the Yellow River Basin serves as the dependent variable in the model, while the environmental regulation indicators constitute the core independent variables. Additionally, other factors that affect high-quality development are introduced in the form of control variables. To minimize the adverse effects of heteroscedasticity and multicollinearity, each variable is log-transformed following existing literature. The specific baseline model is constructed as shown in Eq (1):

$${lnHd}_{it}=\alpha_{0}+\alpha_{1}\;\ln\;{Er}_{it}+\beta_{1}ln{Cons}_{it}+\varepsilon_{it}$$ (1)

In the Eq (1), *i* represents the province (*i* = 1,2,⋯,9), *t* represents the year (t = 2011,2012, …., 2022), *Hd*_*it*_ denotes the level of high−quality development for each province in the Yellow River Basin for each year, *Er*_*it*_ denotes the intensity of environmental regulation for each province in the Yellow River Basin for each year, *Cons*_*it*_ represents the control variables, and *ε*_*it*_ is the stochastic disturbance term.

1. High-quality Economic Development (*Hd*_*it*_). There is no unified standard in academia for measuring the high-quality development of the economy. In the early stages, some scholars used a single Total Factor Productivity (TFP) indicator to measure the high-quality growth of specific regions, which could not fully reflect the connotations of high-quality development and could not meet the demands for quality. This paper, referring to existing research [54] and in line with China’s new development concept strategy—namely innovation, coordination, green, openness, and sharing—along with the evaluation indicators in "Made in China 2025", constructs a high-quality development evaluation index system that includes 29 basic indicators, as shown in Table 1.
2. Environmental Regulation Intensity (*Er*). The academic community’s various methods for measuring environmental regulation can be summarized as single-indicator measurement methods and multidimensional comprehensive methods. Both methods have their characteristics and applicability: the single-indicator measurement method is relatively convenient in data collection, especially showing its convenience when assessing the impact of environmental regulation on specific enterprises or industries, but relying on a single indicator may lead to an imprecise assessment of the strength of environmental regulation. For instance, Low and Yeats [55] proposed in 1992 to assess the intensity of environmental regulation based on the number of regulations issued by the government. Cole and Elliott [56] in 2003 used the emission density of different pollutants as a measurement criterion. In their 2003 study, Aiken and Pasurka [57] took the ratio of operational costs for industry pollution control to industrial output as an indicator of measurement. In contrast, the multidimensional comprehensive method integrates multiple indicators, thereby improving the accuracy of the assessment. Given that the research subjects of this paper are provincial-level samples, it is more appropriate to choose the multidimensional comprehensive method for analysis. At the same time, for the convenience of the study, drawing on existing research [58, 59], this research uses the reciprocal of the pollution comprehensive index as the standard for measuring environmental regulation, with the specific formulas as shown in Eq (2) and Eq (3). The level of this indicator reflects the degree of government effort in pollution control; the higher the index, the greater the control effort, and vice versa, indicating insufficient effort.
3. Control Variables. The process of high-quality economic development is also influenced by factors such as the economic development level of each province, human capital, the degree of marketization, and the urbanization rate. Therefore, the control variables respectively select per capita regional gross domestic product (*Pgp*) to measure the level of economic development, average number of students enrolled in colleges and universities per 100,000 population (*Huc*), the proportion of actual foreign investment in the current year’s industrial fixed assets to measure the degree of marketization (*Dom*), and the proportion of urban population (*Urb*) to measure the level of urbanization.

**Table 1:** Comprehensive index for high-quality economic development in the Yellow River Basin.

First index	Second index	Indicator Connotation	Attribute
Innovation	Per Capita Patent Authorizations	Number of patent authorizations per capita at the end of the year	+
	R&D Investment Ratio	Expenditure on research and experimental development as a share of GDP	+
	GDP Growth Rate	The real GDP growth rate adjusted for inflation	+
Coordination	Regional Income Sharing Level	Sample province per capita GDP / National per capita GDP	+
	Regional Consumption Sharing Level	Sample province residents per capita consumption expenditure / National per capita consumption expenditure	+
	Urban-Rural Income Coordination Level	Per capita disposable income of urban residents / Per capita disposable income of rural residents	+
	Urban-Rural Consumption Coordination Level	Per capita consumption expenditure of urban residents / Per capita consumption expenditure of rural residents	+
Green	hemical Oxygen Demand (COD) Emission per Unit GDP	COD emissions in wastewater / Regional gross domestic product	-
	Waste Gas Emission per Unit GDP	Sulfur dioxide emissions / Regional gross domestic product	-
	Solid Waste Emission per Unit GDP	General industrial solid waste generation / Regional gross domestic product	-
	Greening Coverage Rate	Forest coverage rates of each province	+
	Pollution control	Expenditure on environmental protection in provincial finances / General budgetary expenditure in provincial finances	+
Openness	Foreign Trade Openness	Total value of import and export trade / GDP	+
	Foreign Investment Openness	Actual amount of foreign direct investment utilized / GDP	+
	International Tourism Reception	Number of inbound overnight tourists received	+
	Number of Foreign-Invested Enterprises	Number of foreign-invested enterprises	+
sharing	Number of College Students per 100,000 People	Average number of college students per 100,000 people	+
	Unemployment Rate	Registered urban unemployment rate	-
	Bed Rate per 10,000 People in Medical and Health Institutions	Number of beds in medical and health institutions / Permanent population	+
	Per Capita Highway Mileage	Per capita highway mileage	+

Note: The "+" (or "-") in the Attribute column indicates that under the set measurement method, the indicator is a positive (or negative) indicator, with larger (or smaller) values being more (or less) favorable.


$${Em}_{ipt}=\frac{e_{ipt}}{\sum_{1}^{n}e_{ipt}}/\frac{y_{it}}{\sum_{1}^{n}y_{it}}$$ (2)

$${Er}_{it}=3/\sum_{1}^{3}{Em}_{ipt}$$ (3)

In Eqs (2) and (3), *e*_*ipt*_ refers to the total emissions of a specific pollutant (Pth), *y*_*it*_ refers to the value added of industrial activity, and *Er*_*it*_ is the indicator used to measure the intensity of environmental regulation in each province.

The Environmental Kuznets Curve suggests that as economies and living standards grow, the demand for a healthy environment can exceed that for economic growth. China’s strategy for ecological civilization has made ecological construction a key performance metric for officials, driving a desire for high-quality development and stronger environmental regulations. This can lead to endogeneity issues in the relationship between environmental regulation and economic development, conflicting with OLS estimation assumptions. To address endogeneity and multicollinearity, this paper uses an instrumental variable least squares regression model, following established research methods, to ensure more consistent and reliable results.


$$ln{Er}_{it}=\beta_{0}+\beta_{1}ln{IV}_{it}+\vartheta_{1}ln{Cons}_{it}+e_{it}$$ (4)

$$ln{Hd}_{it}=\delta_{0}+\delta_{1}\;\ln\;{Er}_{it}+\vartheta_{2}ln{Cons}_{it}+\varepsilon_{it}$$ (5)

In the model, *IV*_*it*_ represents the instrumental variable, and *e*_*it*_ is the stochastic disturbance term, with all other variables being the same as in Eq (1). Since rainfall (*Rf*) is significantly negatively correlated with the pollution levels of each province—meaning that areas with higher rainfall tend to have lower pollution levels and are more inclined to adopt less stringent environmental regulations—rainfall is often used as an instrumental variable, drawing from existing research [60]. Rainfall is clearly exogenous to high-quality economic development and affects it only through environmental regulation. Rainfall data is collected from the China Meteorological Administration’s website.

#### 3.1.2 Heterogeneity of pollution levels in the Yellow River Basin

To examine the differences in the impact of environmental regulation on high-quality economic development under the condition of regional pollution heterogeneity, this study draws on existing research and employs the Entropy-Weighted TOPSIS method to construct the pollution index for the Yellow River Basin as shown in Table 2. It uses this index to categorize the pollution heterogeneity of the nine sample provinces in the Yellow River Basin. The study selects the average emission levels of the three types of industrial waste (including industrial wastewater, SO_2_, and dust emissions, with weights of 1/3 each) from each province to calculate the pollution index of the Yellow River Basin. To eliminate the influence of regional industrial scale, the emission amount of pollutants per unit of industrial value added is used to measure the average value of pollution levels for the sample years. This index reflects the amount of pollutant emissions per unit of industrial value added, making it a negative indicator—higher values indicate greater levels of regional pollution.

**Table 2:** Indicators for heterogeneity division of pollution areas in the Yellow River Basin.

Primary Indicator	Secondary Indicator	Weight
Index of Pollution in the Yellow River Basin	Effluent discharge per unit industrial added value	1/3
	Sulfur dioxide (SO_2_) emissions per unit industrial added value	1/3
	Dust emissions per unit industrial added value	1/3

#### 3.1.3 Data sources

The data used in this paper mainly comes from the China Economic Network Statistical Database, China Statistical Yearbook, China Energy Statistical Yearbook, China High-tech Industry Statistical Yearbook, China Environmental Statistical Yearbook, National Economic and Social Development Statistical Bulletin, and National Educational Development Statistical Bulletin. This study takes the data from 2011 to 2021 of the 9 provinces in the Yellow River Basin of China as the research sample. Missing data is supplemented by linear interpolation, and value data is deflated to the base period of 2011. Descriptive statistics of the sample are shown in Table 3.

**Table 3:** Descriptive statistics of concerning variables.

variables			Mean	SD.	Max.	Min.	Obs.
Explained variable	*lnHd*	High-quality economic development index	0.3659	0.1101	0.6767	0.2774	99
Explanatory variable	*lnEr*	Environmental regulation index	0.4001	0.2781	0.9501	0.0475	99
Control variables	*lnPgp*	The level of economic development	2.3711	0.0285	2.4324	2.2984	99
	*lnHuc*	Human capital	2.0772	0.1977	2.3970	1.6432	99
	*lnDom*	Degree of marketization	3.4962	0.6752	5.3861	2.6701	99
	*lnUrb*	Urbanization level	-0.6148	0.1356	-3.826	-0.9875	99
Instrumental variable	*lnRf*	average annual rainfall	-6.3146	0.7806	-5.0131	-9.3627	99

it can be observed that during the sample period, the mean value of the high-quality economic development index (lnHd) for the sample provinces is -0.8247, with a standard deviation of -2.3177, a minimum value of -1.2823, and a maximum value of -0.3905. This indicates that there is a significant difference in the high-quality economic development index among the provinces during the research period. The explanatory variable, environmental regulation (lnEr), also exhibits similar characteristics, which meet the statistical basis for subsequent model regression.

### 3.2 Empirical research results analysis

#### 3.2.1 High-quality development evaluation

Based on the calculated data, the high-quality development assessment results of the nine provinces in the Yellow River Basin from 2011 to 2021 show a positive growth trend. The high-quality development index of all provinces has been increasing year by year, indicating that the development quality in economic, social, and environmental aspects of these areas has been continuously improving. Ningxia, Gansu, Qinghai, Shanxi, and Inner Mongolia have shown relatively stable growth trends, with the index values increasing slightly year by year, demonstrating a steady pace on the path of high-quality development in these regions. Especially in Inner Mongolia, there was a significant improvement from 0.2968 in 2011 to 0.3375 in 2021, reflecting that the region may have achieved important accomplishments in environmental governance and industrial upgrading.

Shaanxi and Sichuan have slightly faster growth rates, with Sichuan in particular seeing its high-quality development index rise from 0.3556 in 2011 to 0.3882 in 2021, which may imply that the province has made significant progress in innovation-driven development and industrial structure optimization. Henan and Shandong have a significantly higher high-quality development index than other provinces, especially Shandong, which has continued to lead with an index increase from 0.6360 in 2011 to 0.6767 in 2021, possibly reflecting its outstanding performance in economic structural adjustment, technological innovation, and the construction of ecological civilization.

Overall, these data reflect that the provinces in the Yellow River Basin have achieved positive effects in pursuing high-quality development. Each region, based on its own characteristics and advantages, has promoted the optimization and upgrading of economic structure and the enhancement of sustainable development capabilities. In the future, these provinces should continue to deepen reform and opening up, strengthen regional collaboration, and promote green transformation to achieve more balanced, inclusive, and sustainable high-quality development.

#### 3.2.2 Pollution level assessment

Based on the assessment results of pollution levels, an average was taken of the pollution levels for the sample years in the Yellow River Basin. The heterogeneity division results of pollution levels for the 9 sample provinces are as follows: (1) Light Pollution: Qinghai Province, Ningxia Hui Autonomous Region, Gansu Province. (2) Moderate Pollution: Shandong Province, Shaanxi Province, Sichuan Province. (3) Severe Pollution: Shanxi Province, Inner Mongolia Autonomous Region, Henan Province.

#### 3.2.3 Benchmark regression analysis

Prior to the panel data regression analysis, a statistical test was conducted to select the appropriate model. The Hausman test statistic revealed that the fixed effects model is more effective than the random effects model. Consequently, the fixed effects model was selected to estimate the impact of environmental regulation on the high-quality economic development in the Yellow River Basin. The regression results are presented in Table 4. Result (1) displays the results for the entire sample of the Yellow River Basin. Results (2), (3), and (4) correspond to the regression outcomes for the light pollution group, moderate pollution group, and severe pollution group, respectively. The findings indicate that environmental regulation exerts a positive influence on the process of high-quality economic development in the Yellow River Basin. The estimated coefficient for environmental regulation is 0.1072, which is statistically significant at the 1% level. This suggests that a 1% increase in the stringency of environmental regulation can effectively lead to a 0.1072% increase in high-quality economic development. This finding aligns with the Porter Hypothesis scenario. Enterprises, in an effort to comply with regulations and reduce environmental costs, adopt advanced environmental technologies, thereby positively influencing the transition to high-quality development, thus confirming H1.

**Table 4:** Baseline regression results.

	(1)	(2)	(3)	(4)
	Global sample regression	low pollution group	medium pollution group	high pollution group
*lnEr*	0.1072**	0.1033	0.1087	0.1182***
	(0.0940)	(0.1423)	(0.1107)	(0.1077)
*lnPgp*	0.4908***	0.6335*	0.5219	0.4367***
	(0.0982)	(0.0522)	(0.1824)	(0.0929)
*lnHuc*	0.1242**	0.2502	0.3025*	0.4233**
	(0.0581)	(0.0950)	(0.0185)	(0.0524)
*lnDom*	0.0117*	0.0052	0.0960**	0.0141***
	(0.0364)	(0.0175)	(0.0854)	(0.0854)
*lnUrb*	-0.9851***	-1.1724*	-1.0521	-0.6630***
	(0.3854)	(0.5214)	(0.5487)	(0.2451)
_*cons*	-2.1465	-3.5194	-3.1805**	-2.9510
	(0.8542)	(1.0527)	(0.8054)	(0.9824)
*observations*	99	33	33	33
*R*−*squared*	0.6071	0.8254	0.8002	0.8651

Note

***, **, and * indicate the significance at the 1%, 5%, and 10% levels respectively, and the t values are in parentheses.

Table 4’s last three columns present the regression results for the Yellow River Basin, grouped by province according to the heterogeneity of pollution levels. In terms of sign, the relationship between environmental regulation and high-quality economic development shows a positive correlation across all groups. However, the regression coefficient is only significant at the 1% level for the high pollution group, indicating that for provinces with severe pollution levels, a 1% increase in the intensity of environmental regulation can lead to a 0.1182% increase in the level of high-quality economic development. Quantitatively, the impact of environmental regulation becomes more pronounced as the level of pollution increases. These regression results are in line with observed reality, where the relationship between environmental regulation and the level of high-quality development varies depending on the degree of pollution. These findings also validate H1. Lanoie et al. [61], using the manufacturing sector in Quebec as an example, demonstrated that sectors with more severe pollution and more intense international competition are more in line with the Porter Hypothesis, which is similar to H1 in this paper.

The regression results for the control variables are interpreted as follows:

Firstly, the regression results for both the overall sample and the subgroup samples indicate that the level of economic development has a significant positive impact on the high-quality economic development of the Yellow River Basin. The regression results for the light pollution group are greater than those for the heavy pollution group, while the moderate pollution group is not significant. Relatively speaking, the economic development level of the light pollution group has a greater driving effect on high-quality economic development. Talent is the key to driving high-quality development. In areas with severe pollution, to mitigate environmental impacts, businesses and governments tend to invest in clean technologies, which increases the demand for professionals with specialized skills and enhances the importance of human capital. Governments may strengthen environmental regulations and improve emission standards in response to high pollution levels, encouraging enterprises to adopt advanced technologies and management strategies, which relies on talents who have received quality education. Public awareness of environmental issues is deeper in areas with severe pollution, which may influence their educational and career choices, inclining them to select fields that contribute to environmental protection and sustainable development, thereby affecting the distribution of human capital.

Secondly, the regression results for both the overall sample and the subgroup samples indicate that the level of human capital has a significant positive impact on the high-quality economic development of the Yellow River Basin. In terms of the significance of the regression coefficients, only the impact of human capital level on high-quality economic development in the light pollution group is not significant. In terms of the magnitude of the regression coefficients, the impact coefficient of human capital level on high-quality economic development in the moderate pollution group is lower than that in the heavy pollution group. The possible reason is that the heavy pollution group pays more attention to human capital investment, and the investment effect has a significant promoting effect on high-quality economic development. As the level of pollution improves, the dependence of high-quality economic development on human capital will decrease.

Thirdly, the regression results for both the overall sample and the subgroup samples indicate that the degree of marketization has a significant positive impact on the high-quality economic development of the Yellow River Basin. In terms of the significance of the regression coefficients, similar to human capital, only the light pollution group is not significant. On the one hand, in areas with moderate and heavy pollution, the improvement of market openness may promote technological innovation and industrial upgrading. The opening of the market introduces international competition, forcing local enterprises to improve production efficiency and product quality, while absorbing and adopting advanced technology and management experience, thereby promoting the transformation of the economy towards high-quality development. On the other hand, market opening may be accompanied by stricter environmental regulation, prompting enterprises to adopt more environmentally friendly production methods and reduce pollution emissions. This is in line with the concept of green development and helps to achieve a win-win situation for economic development and environmental protection.

Fourthly, unlike the impact of other control variables, the urbanization rate generally has a negative impact on the high-quality economic development of the Yellow River Basin. With the acceleration of urbanization, a large influx of people into cities may exert significant pressure on urban resources and the environment, such as water shortages, increased energy consumption, air pollution, and other issues, which may restrict the high-quality development of the economy. In addition, the regression results also show that the promotion of the urbanization rate in provinces with lower pollution intensity has a greater negative impact on high-quality economic development. The possible reason is that provinces with lower pollution levels may have more economic structures dominated by the service industry or high-tech industries, which have higher requirements for environmental quality. If environmental protection is neglected during the urbanization process, it may affect the competitiveness of these industries.

#### 3.2.4 Endogeneity treatment in the model

Table 5 presents the instrumental variable regression results, where result (1) represents the benchmark regression results, and results (2) and (4) are the two-stage least squares regression results. The regression analysis of the first stage shows that the calculation result using rainfall as an instrumental variable is statistically significant at the 5% significance level, effectively ruling out the potential problem of weak correlation that might exist with the instrumental variable. In the first stage of estimation, the coefficient for rainfall is -0.1033. This statistical indicator reveals a significant negative correlation between rainfall and environmental regulation. This implies that in areas with higher rainfall, due to the ease with which pollutants can naturally disperse and decompose, these areas may implement more lenient environmental regulatory measures. Conversely, in areas with less rainfall, due to the lack of natural purification effects, there may be a tendency to adopt stricter environmental regulatory measures to compensate for the deficiency in natural purification capabilities, which aligns with theoretical expectations. The second-stage estimation results show that the estimated coefficient for environmental regulation is 0.0741 and passes the significance test at the 5% level. Compared with the benchmark regression model, the significance of the coefficient for environmental regulation in the second-stage regression is consistent, although the value of the coefficient is lower, it does not change the robustness of the basic regression results. This eliminates the impact of endogeneity, and the model setting and the choice of instrumental variables meet the requirements.

**Table 5:** Regression results based on instrumental variables.

	(1)	(2)	(3)
	Baseline regression	First stage regression	Second stage regression
Explained variable	*LnHd*	*LnEr*	*LnHd*
*lnEr*	0.1072**		0.0741**
	(0.0940)		(0.0460)
*lnRf*		-0.1033**	
		(0.1423)	
*lnPgp*	0.4908***	0.7159***	0.4908
	(0.0982)	(0.1011)	(0.0751)
*lnHuc*	0.1242**	0.5022	0.1038*
	(0.0581)	(0.0981)	(0.0394)
*lnDom*	0.0117*	0.0102	0.0933**
	(0.0364)	(0.0150)	(0.0127)
*lnUrb*	-0.9851***	-1.0214*	-1.0186
	(0.3854)	(0.5084)	(0.2017)
_*cons*	-2.1465	-4.3519	-2.0109**
	(0.8542)	(0.8973)	(0.6028)
*observations*	99	99	99
*R*−*squared*	0.6071	0.6851	0.7546

Note

***, **, and * indicate significance at the 1%, 5%, and 10% levels respectively, and the t values are in parentheses.

Table 6 shows the results of the re-regression using instrumental variables based on pollution heterogeneity. Results (1), (4), and (7) are the benchmark regression results, while Results (2), (5), and (8) represent the first-stage regression results. Results (3), (6), and (9) are the second-stage regression results. The first-stage regression results indicate that the heterogeneity characteristics of pollution levels do not affect the negative impact of the instrumental variable on environmental regulation, which is in line with the actual situation. Compared with the benchmark regression, the second-stage regression results show that the positive impact of environmental regulation in the light pollution group changes from insignificant to significant, and the coefficient decreases from 0.2056 to 0.0894. This suggests that there is an endogeneity issue in the light pollution group samples, and the positive effect of environmental regulation may have been overestimated, which is consistent with the actual situation. In areas with light pollution, there is usually better ecological resilience, a greater number of high-tech enterprises and research institutions, and stronger technological innovation capabilities, making the high-quality development model more likely to gain social and governmental recognition and support. Furthermore, the significance of the regression results for the moderate and severe pollution groups does not change, and the change in the coefficients is small, indicating that the basic regression results are robust and free from endogeneity influence.

**Table 6:** Instrumental variable estimation regression results considering heterogeneity of pollution levels.

	(1)	(2)	(3)	(4)	(5)	(6)	(7)	(8)	(9)
	*LnHd*	*lnEr*	*LnHd*	*LnHd*	*lnEr*	*LnHd*	*LnHd*	*lnEr*	*LnHd*
*lnEr*	0.2056		0.0894***	0.1028		0.0858	0.3328***		0.2004***
*lnRf*	-1.8201*	-1.9033***		-0.5011**	-0.4932***		-0.9651**	-1.0035***	
*lnPgp*	0.7536***	0.9054***	0.2364***	1.3359***	1.2056***	0.5628	1.5294**	1.8543***	-0.0877
*lnHuc*	0.4082**	0.2982	0.3008***	1.5619**	1.0382***	0.8836***	0.1108	0.0972	0.0602***
*lnDom*	0.0836	0.1057	0.0560	0.6021***	0.4957***	0.2059	-0.2276*	-0.3358***	0.1026***
*lnUrb*	1.1775***	1.3378***	-1.0028***	-5.0581***	-4.3691***	-1.5024***	-3.5973*	-2.0954***	0.1157
_*cons*	-6.0213***	-5.0210**	-4.0951	-4.2219**	-4.9824***	-5.3391***	-4.3050	-3.2546	0.8247
*obs*.	33	33	33	33	33	33	33	33	33
*R* ^2^	0.7521	0.7820	0.8055	0.7729	0.8127	0.7851	0.7273	0.7528	0.8044

Note

***, **, and * indicate significance at the 1%, 5%, and 10% levels respectively.

#### 3.2.5 Non-linear regression analysis

The relationship between environmental regulation and economic development is often complex and not simply linear. Different regions with varying pollution levels may respond differently to environmental regulations. Areas with light pollution may more readily adapt to and leverage environmental regulations to promote high-quality development, while areas with heavy pollution may face greater pressure to transform. By examining non-linear effects, we can more accurately assess the effectiveness of environmental regulation policies, providing a basis for policy adjustment and optimization. To this end, the quadratic term of environmental regulation is added to Eq (1). The Hausman test result indicates that the fixed effects model is optimal. Results (1)-(4) in Table 7 show the regression results for the overall Yellow River Basin sample group, light pollution group, moderate pollution group, and heavy pollution group, respectively.

**Table 7:** Regression results of nonlinear effects under heterogeneous pollution levels.

	(1)	(2)	(3)	(4)
	Full group	Light group	Medium group	Heavy group
*lnEr*	0.2518***	0.3201	0.0641	0.2719***
	(0.0684)	(0.3751)	(0.0591)	(0.0684)
*lnEr* ^2^	0.3594***	0.0684*	0.1914**	0.4516*
	(0.0772)	(0.2051)	(0.4029)	(0.0614)
*lnPgp*	0.5527*	0.6028***	0.3851	0.0814
	(0.2154)	(0.1128)	(0.1549)	(0.0324)
*lnHuc*	0.0682***	0.0284	0.1549*	-0.0014*
	(0.0647)	(0.1028)	(0.0257)	(0.0210)
*lnDom*	0.0621*	0.0330	0.1128***	-0.0518
	(0.0681)	(0.2059)	(0.0519)	(0.0027)
*lnUrb*	-1.0242***	-1.3846	-1.9547	0.0018
	(0.6218)	(2.0051)	(0.8438)	(0.1958)
_*cons*	-1.2846**	-3.0054***	-3.8543	-1.2154*
	(0.9514)	(1.7721)	(0.8614)	(0.3318)
*obs*.	99	33	33	33
*R* ^2^	0.6841	0.8514	0.8057	0.9201

Note

***, **, and * indicate significance at the 1%, 5%, and 10% levels respectively.

The model estimation results show that the estimated coefficients for the quadratic term of environmental regulation are positive and significant for all sample groups, thus validating Hypothesis 2. There is a considerable variation in the estimated coefficients of the environmental regulation quadratic term across the sample groups, indicating that the non-linear impact of environmental regulation on high-quality economic development also varies significantly depending on the level of pollution in different regions. In areas with higher levels of pollution, the positive effect of environmental regulation on the process of high-quality economic development has the largest coefficient.

#### 3.2.6 Threshold effect test

The previous section has demonstrated that the impact of high-quality economic development on environmental regulation has non-linear characteristics. To further analyze the specific thresholds of non-linear effects, as well as differences between provinces with heterogeneous pollution levels, a panel threshold model is constructed by introducing environmental regulation as a threshold variable. Before estimating the threshold model, a panel threshold existence test was first conducted based on the method of Hansen (1999) [62]. After using the "bootstrap" method with repeated sampling 3000 times, the results indicate that the impact of environmental regulation on high-quality economic development for the overall sample group, light pollution group, moderate pollution group, and heavy pollution group all significantly passed the single threshold test and did not pass the double threshold and triple threshold tests, as shown in Table 8. On this basis, a single threshold model with environmental regulation as the threshold variable was set up, as shown in Eq (6), and the regression results were obtained as shown in Table 9.


$${lnHd}_{it}=\rho_{0}+\sigma_{1}\;\ln\;{contrs}_{it}+\pi_{1}\;\ln\;{Er}_{it}*I(\ln\;{Er}_{it}\leq \tau_{1})+\pi_{2}\;\ln\;{Er}_{it}*I(\ln\;{Er}_{it}>\tau_{1})+\varepsilon_{it}$$ (6)

**Table 8:** Environmental regulation panel threshold model test.

Group	Threshold test	Threshold	F	P	10%	5%	1%
Full Group	single	0.2334*	32.7784	0.0721	19.6742	34.9720	42.6397
	Double	0.3761	6.6847	0.5886	13.6592	20.6893	28.5286
Light Group	single	0.3933***	14.4587	0.0007	8.1859	9.3295	10.6840
	Double	0.7317	7.1153	0.1734	16.2748	30.5528	38.9362
Medium Group	single	0.3077**	9.0648	0.3857	6.8462	8.4406	11.5271
	Double	0.4417	5.4648	0.2758	8.3852	11.7392	18.6309
Heavy Group	single	0.1362***	11.3981	0.0002	8.1106	9.0371	10.3827
	Double	0.1847	10.3827	0.1288	14.3859	19.7704	24.1990

Note: The Bootstrap method is used to repeat sampling 5000 times to obtain the P value and critical value.

**Table 9:** Regression results of panel threshold model.

	Full sample	Light pollution	Medium pollution	Heavy pollution
*lnEr*	-0.0509	-0.5016***	0.0117*	0.0301***
	(0.0524)	(0.1953)	(0.0911)	(0.1044)
lnEr*r_1_	0.1543***	-0.0103	-0.1027**	-0.1160***
	(0.0602)	(0.2054)	(0.2054)	(0.2008)
lnEr*r_2_	0.5109*	0.3086	0.0084**	0.6927***
	(0.0754)	(0.2046)	(0.2046)	(0.0706)
*lnPgp*	0.1011**	0.1618***	0.1137***	0.0921
	(0.0758)	(0.0953)	(0.0546)	(0.0692)
*lnHuc*	0.1683	0.3015**	0.1524*	0.0901*
	(0.0510)	(0.1953)	(0.0957)	(0.0209)
*lnDom*	0.0503*	0.0652**	0.0485**	0.0205***
	(0.0299)	(0.0468)	(0.0354)	(0.0704)
*lnUrb*	-1.3085***	-1.4824***	-0.8461*	-0.2567*
	(0.4007)	(0.6024)	(0.3884)	(0.2549)
_*cons*	-1.8851	-3.0057	-2.9124	-2.5186
	(0.5691)	(3.5814)	(2.3541)	(0.4461)
*obs*.	99	33	33	33
*R* ^2^	0.7046	0.8791	0.8907	0.9204

In the Eq 6, *contrs*_*it*_ represents the set of control variables, *I*(*) is the indicator function, *τ*_1_ is the threshold value, and *ε*_*it*_ is the stochastic disturbance term.

The threshold regression outcomes are displayed in Table 9. For the comprehensive sample of the Yellow River Basin, environmental regulation is categorized into two ranges based on the threshold value: *lnER* ≤ 0.2334 and *lnER* > 0.2334. At *lnER* ≤ 0.2334, the coefficient for environmental regulation is 0.1543, significantly passing the test at the 1% level, signifying a positive and promoting impact of environmental regulation on high-quality economic development within the initial threshold range. Beyond *lnER* > 0.261, the coefficient escalates to 0.5109, which is notably significant at the 10% level, suggesting that heightened environmental regulation intensity is beneficial in fostering high-quality economic development.

For the light pollution sample of the Yellow River Basin, environmental regulation is divided into two intervals based on the threshold value: *lnER* ≤ 0.3993 and *lnER* > 0.3993. When *lnER* ≤ 0.3993, the effect of environmental regulation on promoting high-quality economic development is negative, though not significant. This implies that the light pollution group is not suited to an excessively low intensity of environmental regulation, as it could hinder high-quality economic development to some extent. When *lnER* > 0.3993, the estimated coefficient for environmental regulation turns positive from negative, and, while not passing the significance test, it still indicates that once the intensity of environmental regulation reaches a certain level, it can have a positive effect on promoting high-quality economic development in the light pollution group.

For the moderately polluted sample of the Yellow River Basin, environmental regulation is divided into two intervals based on the threshold value: *lnER* ≤ 0.3077 and *lnER* > 0.3077. When *lnER* ≤ 0.3077, the effect of environmental regulation on promoting high-quality economic development is negative and significant at the 5% level. This indicates that for the moderately polluted group, an excessively low intensity of environmental regulation will hinder high-quality economic development to a certain extent. When *lnER* >0.3077, the estimated coefficient for environmental regulation turns positive from negative and passes the significance test at the 5% level. This suggests that once the intensity of environmental regulation reaches a certain threshold, it can positively promote high-quality economic development in the moderately polluted group.

For the severely polluted sample group of the Yellow River Basin, environmental regulation is divided into two intervals based on the threshold value: *lnER* ≤ 0.1362 and *lnER* > 0.1362. When *lnER* ≤ 0.1362, the effect of environmental regulation on high-quality economic development shows a significant negative effect; when *lnER* > 0.1362, the impact shifts to a significant positive effect, with a substantial increase in the coefficient. This indicates that a higher intensity of environmental regulation can more effectively promote high-quality economic development in severely polluted areas.

Integrating the aforementioned regression results, it can also be observed that for samples with lower pollution levels, a stronger environmental regulation is superior to a lower intensity of environmental regulation. Among the three subgroups, as the intensity of environmental regulation increases, there is a point at which the impact of environmental regulation on high-quality economic development shifts from negative to positive, dividing its effect on high-quality economic development into two parts, akin to a "V-shaped" relationship. Comparatively, in the regression results of the overall Yellow River Basin sample, the impact of environmental regulation on high-quality economic development has always been positive, and as the intensity of environmental regulation increases, its influence on high-quality economic development also becomes greater.

Integrating the analysis of existing literature, there is a significant variation in the non-linear relationship between environmental regulation and high-quality development. The reason may primarily be due to the differences in the selection of research samples and time periods. The theoretical analysis of **H1** suggests that only an "appropriate" level of environmental regulation can promote high-quality economic development, while the promotion of high-quality economic development by environmental regulations that are too strong or too weak needs to be proven through testing.

### 3.3 Robustness test

In the previous sections, heterogeneity issues have been thoroughly discussed. The following robustness checks are conducted from two perspectives: alternative explanatory variables and adjustment of the sample interval.

It is a common method for robustness testing to use the fact that different indicators of environmental regulation intensity are somewhat correlated and have similar theoretical bases. This paper draws on existing research and uses the inverse of industrial wastewater discharge intensity as an alternative indicator of environmental regulation to re-perform panel regression on the model. According to the Hausman test results, the fixed effects model is appropriate for the model. The results of the regression are shown in results (1) and (2) of Table 10. The regression results show that the new environmental regulation variable still significantly affects high-quality economic development in a positive manner, and the coefficient of the quadratic term remains positive and significant. The control variables have changed in terms of the value and significance of the regression coefficients, but the direction of their effect on high-quality economic development has not changed. Overall, by changing the core explanatory variable, it can be proven that the aforementioned research conclusions still hold true, passing the robustness test.

**Table 10:** Regression results of robustness test.

	Changing the measurement of *Er*		Changing the sample sampling interval	
	Linearity test	Nonlinear test	Linearity test	Nonlinear test
	(1)	(2)	(3)	(4)
*lnEr*	0.2033***	0.5895***	0.1547***	0.2070***
	(0.0591)	(0.1788)	(0.042)	(0.0484)
*lnEr* ^2^		0.0772***		0.2162***
		(0.0097)		(0.0489)
*lnPgp*	0.3514*	0.4582*	0.4827**	0.5079*
	(0.1680)	(0.2057)	(0.2087)	(0.3057)
*lnHuc*	0.2547**	0.0880*	0.2038***	0.2043**
	(0.0591)	(0.0572)	(0.0812)	(0.0524)
*lnDom*	0.0751*	0.0648	0.0542*	0.0712
	(0.0218)	(0.0541)	(0.0647)	(0.0617)
*lnUrb*	-0.9840**	-2.0005***	-1.3361*	-1.5027*
	(0.6084)	(0.3281)	(0.5681)	(0.6628)
_*cons*	-5.3367*	0.2467	-4.3954*	-2.5843
	(0.7928)	(0.5008)	(1.1856)	(0.8947)
*obs*.	99	99	99	99
*R* ^2^	0.8001	0.5214	0.5691	0.7001

Additionally, considering that China experienced the outbreak of COVID-19 in 2019, which severely impacted economic development and environmental regulation as an exogenous shock, the data from 2019 to 2021 were excluded to re-estimate the model. According to the Hausman test results, the fixed effects model is still suitable for the model. The regression results are shown in results (3) and (4) of Table 10. The test results indicate that the linear and non-linear effects of environmental regulation on high-quality economic development are robust. At the same time, changes in the control variables are only reflected in the magnitude of the values and their significance, without any significant change in the direction of their impact, which confirms that the relevant research conclusions still hold.

## 4 Discussion-based conclusions

This paper constructs a theoretical analysis foundation and model based on the perspective of pollution heterogeneity, and takes the Yellow River Basin in China as a sample to measure variables such as high-quality development and environmental regulation. It examines the impact of environmental regulation on high-quality economic development, tests the differences in impact under the background of pollution heterogeneity, explores the non-linear relationship between the two, and analyzes the selection issue of environmental regulation intensity, reaching the following conclusions:

Firstly, the level of high-quality economic development in the Yellow River Basin has been fluctuating upwards from 2011 to 2022, with environmental policies promoting the integration of ecological civilization concepts into urbanization. Shandong Province has consistently led other provinces in the region in terms of high-quality economic development. Additionally, during the sample period, there has been a trend of fluctuating decline in the degree of environmental pollution across the provinces within the Yellow River Basin, which can be categorized into three levels based on pollution severity.

Secondly, environmental regulation in the Yellow River Basin positively affects high-quality economic development. There is ample literature supporting this conclusion. For example, He [63] validated that, from a general perspective, environmental regulation contributes to promoting the overall quality of economic growth based on panel data from 28 provinces in China from 2000 to 2014. Wang et al. [64] found that stricter environmental regulation helps to improve the ecological efficiency of the paper industry in Shandong Province. Environmental regulations require businesses to reduce pollution and optimize resource use, thereby increasing resource efficiency. To meet these requirements, companies are compelled to innovate technologically, developing and adopting cleaner production technologies. Such innovation not only lessens environmental impact but also enhances productivity, promotes the development of the environmental protection industry, boosts exports, and drives high-quality economic development.

Thirdly, the impact of environmental regulation varies across regions with differing levels of pollution. The varying influence of environmental regulation in areas with different pollution levels is the result of a combination of factors. These factors may include the degree of pollution, economic development level, human capital level, market openness, and urbanization level of the sample regions. Some literature has obtained results similar to those of this paper. For instance, Feng et al. [65] used panel data from 45 major cities in the Yangtze River Economic Belt in China from 2013 to 2020 and confirmed that an increase in both official and unofficial environmental regulations can promote the improvement of environmental quality. Moreover, the impact of environmental regulation is greater in cities with better environmental quality, and implementing both official and unofficial environmental regulations simultaneously can better improve environmental quality than implementing a single type of regulation.

Fourthly, in areas with severe pollution, the effect of environmental regulation on promoting high-quality economic development is more pronounced. There is a considerable amount of literature that reaches similar conclusions. For example, Jaffe [40] found that stronger environmental regulation can effectively stimulate an increase in R&D investment. Regions with severe environmental conditions may see more significant results from pollution reduction measures. Moreover, in areas with serious pollution, the public’s awareness of environmental issues is stronger, and their willingness to cooperate with environmental regulations is higher. This public pressure can encourage governments and businesses to adopt environmental regulation measures more actively.

Fifthly, environmental regulation has a non-linear impact and threshold effects on high-quality economic development. For the overall sample, the threshold value is 0.2334. The impact before and after the threshold is positive, indicating that increasing the intensity of environmental regulation is beneficial for promoting high-quality economic development. For the three subgroups, the impact changes from negative to positive before and after the threshold, showing a "V-shaped" relationship between environmental regulation and high-quality development. Overall, in provinces with higher pollution levels, the non-linear positive effect of environmental regulation on the process of high-quality economic development is more pronounced.

## 5 Policy recommendations

Based on the conclusions drawn above, the following policy recommendations are proposed:

Firstly, the level of high-quality development in the Yellow River Basin is not high, and improving it requires comprehensive measures that cover the economy, environment, society, and other aspects. Specifically, in terms of environmental regulation, policies should be formulated according to different pollution levels, tailored to local conditions, to promote the positive impact of environmental regulation and curb negative effects. Enhance the average level of environmental regulation in the Yellow River Basin, promote clean technology innovation and efficient resource utilization, and improve economic benefits. Regions with different levels of pollution require different levels of environmental regulation; a one-size-fits-all policy is not feasible. Instead, suitable environmental regulations should be designed in conjunction with the degree of pollution.

Secondly, establish a regional collaborative governance mechanism, especially for cross-provincial and cross-regional situations, unified planning and management are necessary. On one hand, dynamically adjust the intensity of environmental regulation in various regions based on environmental quality monitoring data and economic development to ensure coordinated progress in environmental protection and economic development. On the other hand, establish a real-time environmental monitoring and early warning system to promptly identify and correct differences in regional environmental regulations and prevent companies from exploiting regulatory loopholes. Strengthen cross-regional environmental law enforcement cooperation, share information and resources for environmental law enforcement, and combat cross-regional environmental violations. Companies that violate environmental regulations should be subject to severe penalties, including fines, production suspension, and legal accountability to increase the cost of violations and curb the pollution haven effect.

Thirdly, enhance public participation and supervision, and improve the legal and regulatory framework. Establish and improve public supervision mechanisms, encourage the public to report environmental violations, and enhance corporate environmental responsibility. Strengthen transparency in environmental information by regularly publishing environmental quality and corporate pollution discharge information to increase transparency in corporate and government environmental governance. Improve laws and regulations related to environmental protection, clarify the environmental protection responsibilities of governments and businesses at all levels, and provide legal protection for environmental regulation. Strengthen environmental judiciary by establishing specialized environmental courts or tribunals to ensure effective implementation of environmental laws and regulations.

Fourthly, promote green technology innovation and establish a pollution trading mechanism. Governments should increase support for environmental protection technology research and development, encourage enterprises to adopt clean production technologies and processes to reduce pollution at the source. Establish green technology demonstration projects to promote successful experiences and advanced technologies, helping enterprises to improve their environmental protection technology level. Establish a pollution trading market within the region to regulate the total amount of pollutant emissions through market mechanisms and encourage enterprises to reduce emissions through technological transformation.

## Supporting information

S1 Appendix (PDF)

S2 Appendix (PDF)
