# Supplementary material for: Towards high-quality development: The complex role of environmental regulation
Source: PLoS One. 2025 Feb 12;20(2):e0312816. doi: 10.1371/journal.pone.0312816 (PMC11819545; doi:10.1371/journal.pone.0312816)
Supplement: S1 Appendix — (PDF) [file pone.0312816.s001.pdf]

TOPSIS model based on the entropy weight method

The entropy value serves as a measure of the variability of indicators, with a lower value indicating a richer amount of information. In terms of weight allocation, the entropy method, compared to principal component analysis, effectively reduces the influence of subjectivity (1). The TOPSIS method ranks evaluation objects based on their closeness to the ideal solution. This approach not only yields reasonable results but also features a simple and intuitive calculation process. By integrating the concept of entropy with the TOPSIS method, a new evaluation approach has been formed. This method combines the strengths of both techniques, enabling a more accurate measurement of the development level of comprehensive indicators. In this study, during the evaluation process of the core variables, relevant literature (2, 3) was referenced, and a TOPSIS model based on the entropy weight method was adopted, striving to ensure that the assessment results of the core variables are characterized by scientific and practical features.

Step 1，The first step is to address the issue that the indicators within the high-quality growth evaluation index system involve different dimensions and numerical scales. To eliminate the impact of these differences, it is a necessary step to dimensionlessly treat the indicators X_{ij}.

$$W_{it} = \begin{cases} \frac{X_{ij} - min(X_{ij})}{max(X_{ij}) - min(X_{ij})}, X_{ij}\ is\ positive\ indicator \\ \frac{max(X_{ij}) - X_{ij}}{max(X_{ij}) - min(X_{ij})}, X_{ij}\ is\ negative\ indicator \end{cases} \quad (1)$$

Step 2，The second step involves quantifying the information content of each indicator through the concept of information entropy. The information entropy of the indicators is measured according to the following formula.

$$E_j = -ln\frac{1}{n}\sum_{j=1}^{m}\left[\left(W_{ij}\right)/\sum_{i=1}^{n}W_{ij}ln\left(W_{ij}/\sum_{i=1}^{n}W_{ij}\right)\right] \quad (2)$$

Step 3，The third step is to assign standardized weights to the indicators, that is, to normalize the weights.

$$G_j = \left(1 - E_j\right)\sum_{j=1}^{m}\left(1 - E_j\right) \quad (3)$$

Step 4，Construction of normalized attribute matrix.

$$R = \left(r_{ij}\right)_{n\times m} r_{ij} = G_j \times W_{ij} \quad (4)$$

Step 5，Determination of positive ideal point Q_j^+ and negative ideal point Q_j^-.

$$Q_j^+ = (maxr_{i1}, maxr_{i2}, \cdots, maxr_{im})$$
$$Q_j^- = (minr_{i1}, minr_{i2}, \cdots, minr_{im}) \quad (5)$$

Step 6, The sixth step is to calculate the distances between each evaluation object and the optimal and worst values.

$$d_i^+ = \sqrt{\sum_{i=1}^{m} \left(Q_j^+ - r_{ij}\right)^2}$$
$$d_i^- = \sqrt{\sum_{j=1}^{m} \left(Q_j^- - r_{ij}\right)^2} \quad (6)$$

Step 7, Calculate the degree of closeness.

$$C_i = \frac{d_i^-}{d_i^+ + d_i^-} \quad (7)$$

Where, the larger the C_i , the higher the high-quality development level of manufacturing in the region.

References:

1. Wang L, Wang Z, Ma Y. Does environmental regulation promote the high-quality development of manufacturing? A quasi-natural experiment based on China's carbon emission trading pilot scheme. Socio-Econ Plan Sci. 2022;81:101216. http://doi.org/https://doi.org/10.1016/j.seps.2021.101216
2. Chen L, Wang Y. Research on TOPSIS integrated evaluation and decision method based on entropy coefficient. Control and Decision. 2003;18(4):456-9.
3. Wang Z, Wang J, Zhang G, Wang Z. Evaluation of Agricultural Extension Service for Sustainable Agricultural Development Using a Hybrid Entropy and TOPSIS Method. Sustainability-Basel. 2021;13(1):1-17. http://doi.org/10.3390/su13010347
