# Supplementary material for: Towards high-quality development: The complex role of environmental regulation
Source: PLoS One. 2025 Feb 12;20(2):e0312816. doi: 10.1371/journal.pone.0312816 (PMC11819545; doi:10.1371/journal.pone.0312816)
Supplement: S2 Appendix — (PDF) [file pone.0312816.s002.pdf]

The evaluation results of the high-quality development level of the Yellow River Basin are shown in Table S2_1:

Table S2-1 Accounting results of high-quality development in the Yellow River Basin

province	2011	2012	2013	2014	2015	2016	2017	2018	2019	2020	2021
neimenggu	0.2968	0.3049	0.3009	0.309	0.3131	0.3172	0.3212	0.3253	0.3294	0.3334	0.3375
gansu	0.2822	0.2863	0.2944	0.2904	0.3067	0.2985	0.3026	0.3107	0.3148	0.3189	0.3230
qinghai	0.2942	0.2860	0.2901	0.2983	0.3064	0.3023	0.3105	0.3146	0.3186	0.3227	0.3268
sichuan	0.3556	0.3474	0.3515	0.3596	0.3637	0.3678	0.3719	0.3759	0.3800	0.3841	0.3882
shandong	0.6360	0.6400	0.6441	0.6482	0.6523	0.6563	0.6604	0.6645	0.6686	0.6726	0.6767
shannxi	0.3073	0.3032	0.3113	0.3154	0.3195	0.3236	0.3276	0.3317	0.3358	0.3399	0.3439
ningxia	0.2815	0.2774	0.2855	0.2937	0.2896	0.2978	0.3018	0.3059	0.3100	0.3141	0.3181
henan	0.4017	0.3935	0.3976	0.4058	0.4098	0.4139	0.4180	0.4221	0.4261	0.4302	0.4343
shanxi	0.2944	0.2863	0.2903	0.2985	0.3026	0.3066	0.3107	0.3148	0.3189	0.3229	0.3270

The results of the Yellow River Basin environmental regulation level assessment are shown in Table S2_2:

Table S2-2 Accounting results of environmental regulation level in the Yellow River Basin

province	2011	2012	2013	2014	2015	2016	2017	2018	2019	2020	2021
neimenggu	0.2212	0.2028	0.2420	0.2596	0.2304	0.2688	0.2580	0.2672	0.3764	0.3856	0.3949
gansu	0.1093	0.1278	0.1001	0.1370	0.1186	0.1554	0.1662	0.1738	0.1846	0.1830	0.2122
qinghai	0.1314	0.1699	0.1791	0.1607	0.1975	0.1883	0.2159	0.2067	0.2251	0.2343	0.2435
sichuan	0.5738	0.5726	0.5854	0.5830	0.5892	0.6106	0.7198	0.7383	0.7314	0.7291	0.8875
shandong	0.8764	0.8672	0.8956	0.8580	0.8748	0.9140	0.9132	0.9225	0.9017	0.9309	0.9501
shannxi	0.3241	0.3325	0.3333	0.3479	0.3387	0.3601	0.3885	0.3893	0.4177	0.4370	0.5562
ningxia	0.0580	0.0565	0.0475	0.0672	0.1049	0.0957	0.1041	0.1225	0.1517	0.1533	0.1909
henan	0.6038	0.5946	0.6354	0.6852	0.6830	0.7166	0.7198	0.7383	0.7514	0.7591	0.8995
shanxi	0.1388	0.1680	0.1672	0.1765	0.1957	0.1949	0.2041	0.2233	0.2425	0.2817	0.3009

The data tables of other variables used in the paper are as follows: Table S2_3~S2_7 (mean value, standard deviation, maximum value and other information of the numbers have been reported in the text of the manuscript) :

Table S2-3 Pgp data in the Yellow River Basin

province	2011	2012	2013	2014	2015	2016	2017	2018	2019	2020	2021
neimenggu	2.3564	2.3661	2.3743	2.3806	2.3867	2.3927	2.3999	2.4074	2.4135	2.4141	2.4324
gansu	2.2869	2.2985	2.3097	2.3180	2.3188	2.3242	2.3301	2.3398	2.3471	2.3501	2.3628
qinghai	2.3121	2.3222	2.3330	2.3399	2.3475	2.3581	2.3656	2.3754	2.3813	2.3829	2.3935
sichuan	2.3195	2.3318	2.3415	2.3494	2.3535	2.3612	2.3733	2.3844	2.3912	2.3950	2.4048
shandong	2.3619	2.3702	2.3789	2.3849	2.3921	2.3969	2.4025	2.4071	2.4119	2.4143	2.4256
henan	2.3260	2.3346	2.3425	2.3505	2.3565	2.3636	2.3731	2.3827	2.3891	2.3896	2.3959
ningxia	2.3335	2.3411	2.3482	2.3527	2.3554	2.3613	2.3731	2.3807	2.3859	2.3902	2.4032
shannxi	2.3406	2.3543	2.3649	2.3728	2.3750	2.3801	2.3905	2.3998	2.4060	2.4065	2.4195
shanxi	2.3347	2.3418	2.3447	2.3458	2.3439	2.3450	2.3634	2.3727	2.3785	2.3833	2.4062

Table S2-4 Huc data of the Yellow River Basin

province	2011	2012	2013	2014	2015	2016	2017	2018	2019	2020	2021
neimenggu	1.9905	2.0027	2.0178	2.0010	2.0274	2.0075	2.0164	2.0247	2.0420	2.0589	2.0709
gansu	2.0014	1.9623	1.9790	1.9875	2.0012	2.0081	2.0183	2.0261	2.0434	2.0609	2.0736
qinghai	1.8594	1.8180	1.8304	1.8418	1.8571	1.8650	1.8758	1.8865	1.9053	1.9270	1.9394
sichuan	2.1349	2.1010	2.2125	2.2327	2.2414	2.2466	2.2546	2.2615	2.2759	2.2900	2.3002
shandong	2.2480	2.2168	2.3273	2.3348	2.3426	2.3482	2.3548	2.3608	2.3758	2.3871	2.3970
henan	2.1483	2.1030	2.2259	2.2332	2.2417	2.2479	2.2555	2.2672	2.2806	2.2913	2.3017
ningxia	1.6432	1.6490	1.6609	1.6777	1.6884	1.7003	1.7160	1.7342	1.7567	1.7741	1.7965
shannxi	2.1617	2.1058	2.2266	2.2368	2.2432	2.2499	2.2579	2.2688	2.2821	2.2930	2.3055
shanxi	2.0657	1.9763	1.9869	1.9968	2.0056	2.0151	2.0233	2.0417	2.0570	2.0689	2.1077

Table S2-5 Dom data of the Yellow River Basin

province	2011	2012	2013	2014	2015	2016	2017	2018	2019	2020	2021
neimenggu	2.6701	2.6763	2.6779	2.7216	2.7648	3.7529	3.8892	3.9069	4.0096	4.1887	4.2931
gansu	2.7701	2.7736	2.8070	2.8503	2.8899	2.9788	3.1023	3.4526	3.6975	3.9316	4.2034
qinghai	2.7233	2.7143	2.7729	2.7890	2.7543	2.8043	2.7143	2.7801	2.7996	2.7657	2.8747
sichuan	3.0821	3.0890	3.0984	3.1272	3.1561	3.2054	3.3199	3.3374	3.5182	3.8326	4.0456
shandong	3.1005	3.4601	3.7233	3.9306	3.4789	3.9669	4.2235	4.5659	4.7239	5.0581	5.3862
henan	3.0701	3.4797	3.7284	3.8860	4.2433	4.2352	4.3533	4.5759	4.7422	4.8815	5.0888
ningxia	2.6767	2.6820	2.7291	3.2291	3.2703	3.3402	3.3904	3.4218	3.4550	3.4653	3.4906
shannxi	3.8212	3.8890	3.9775	3.9370	3.9873	4.0532	4.1960	4.2545	4.3700	4.4281	4.4566
shanxi	2.6861	2.7285	2.7871	2.8393	2.9041	3.0595	3.1045	3.1907	3.2714	3.3613	3.4630

Table S2-6 Urb data of the Yellow River Basin

province	2011	2012	2013	2014	2015	2016	2017	2018	2019	2020	2021
qinghai	-0.6974	-0.6671	-0.6371	-0.6106	-0.5821	-0.5574	-0.5346	-0.5133	-0.4896	-0.4695	-0.4554
gansu	-0.5614	-0.5375	-0.5138	-0.4948	-0.4766	-0.4557	-0.4370	-0.4230	-0.4086	-0.3933	-0.3826
neimenggu	-0.6761	-0.6533	-0.6262	-0.6020	-0.5626	-0.5254	-0.4977	-0.4868	-0.4803	-0.4612	-0.4472
sichuan	-0.9046	-0.8677	-0.8301	-0.7974	-0.7546	-0.7178	-0.6820	-0.6493	-0.6160	-0.5900	-0.5718
shanxi	-0.8711	-0.8359	-0.7994	-0.7655	-0.7284	-0.6931	-0.6582	-0.6255	-0.5913	-0.5669	-0.5478
henan	-0.7476	-0.6990	-0.6622	-0.6347	-0.6026	-0.5729	-0.5435	-0.5167	-0.4897	-0.4674	-0.4521
ningxia	-0.9875	-0.9473	-0.9039	-0.8609	-0.8155	-0.7750	-0.7315	-0.6994	-0.6792	-0.6495	-0.6287
shannxi	-0.7651	-0.7371	-0.7074	-0.6765	-0.6603	-0.6246	-0.5897	-0.5574	-0.5314	-0.5095	-0.4940
shandong	-0.6892	-0.6704	-0.6379	-0.6011	-0.5625	-0.5320	-0.4951	-0.4756	-0.4521	-0.4314	-0.4149

Table S2-7 Rf data of the Yellow River Basin

province	2011	2012	2013	2014	2015	2016	2017	2018	2019	2020	2021
shannxi	-6.4176	-5.9669	-6.8088	-5.9676	-6.2897	-5.8382	-6.4783	-6.0027	-6.6599	-6.5477	-5.9848
shanxi	-7.3276	-6.9346	-7.9114	-8.0177	-6.6535	-7.6174	-8.2530	-9.3627	-7.0396	-7.1222	-7.4462
gansu	-6.8184	-6.8338	-6.6130	-6.7169	-6.6495	-6.8613	-6.5852	-6.6086	-6.6741	-6.7238	-6.6839
sichuan	-5.2956	-5.3573	-5.4407	-5.4298	-5.3279	-5.3067	-5.3800	-5.2426	-5.2560	-5.2859	-5.3104
qinghai	-6.3342	-6.2228	-6.3327	-6.5014	-6.0665	-6.2832	-6.4159	-6.4102	-6.2451	-6.1652	-6.2219
henan	-6.2363	-5.8410	-6.1866	-5.8494	-5.8621	-5.9244	-6.0086	-5.9212	-5.9664	-6.0174	-5.7819
shandong	-5.3023	-5.1709	-5.3140	-5.2167	-5.1691	-5.1723	-5.3061	-5.1002	-5.0131	-5.1760	-5.2208
neimenggu	-6.9511	-6.8924	-6.8401	-6.9140	-6.6656	-6.7784	-6.8261	-6.8495	-6.7716	-6.8657	-6.8312
ningxia	-6.8505	-6.9957	-6.6134	-6.6973	-6.5551	-6.9383	-6.5710	-6.7949	-6.5882	-6.5722	-6.4783
